# Zinc or/and Vitamin E Supplementation Mitigates Oxidative Stress, Neuroinflammation, Neurochemical Changes and Behavioural Deficits in Male Wistar Rats Exposed to Bonny Light Crude Oil

**DOI:** 10.1155/jt/9317271

**Published:** 2024-12-21

**Authors:** Oluwafunmbi Ebenezer Ogunmiluyi, Alexander Obidike Naiho, Victor Oghenekparobo Emojevwe, Tolulope Samuel Oladele, Kayode Adedoyin Adebisi, Jacob Adewale Siyanbade, Adeniran Oluwadamilare Akinola

**Affiliations:** ^1^Department of Physiology, University of Medical Sciences, Ondo, Ondo, Nigeria; ^2^Department of Physiology, University of Delta, Agbor, Delta, Nigeria; ^3^Department of Physiology, Delta State University, Abraka, Delta, Nigeria; ^4^Department of Anatomy, University of Medical Sciences, Ondo, Ondo, Nigeria; ^5^Department of Anatomy, Ladoke Akintola University, Ogbomoso, Oyo, Nigeria

**Keywords:** Bonny crude oil, neurobehaviour, neurochemicals, vitamin E, zinc

## Abstract

**Background:** Crude oil, a major key economic driver in developing countries, is also of environmental concern, linked to neurotoxicity and behavioural problems. Despite the known neurotoxic effects of crude oil and the potential benefits of zinc and vitamin E, there is a paucity of research specifically addressing their combined efficacy in mitigating neurochemical changes and behavioural deficits induced by crude oil. Current studies have largely focussed on the individual effects of these supplements in different contexts, but their synergistic potential in a crude oil exposure model remains underexplored. This study investigated the potential effects of zinc and vitamin E on neurobehavioural alterations in male Wistar rats fed with Bonny light crude oil (BLCO)-contaminated diet.

**Methods:** Thirty (30) male Wistar rats (160 ± 10 g) were assigned into five groups (*n* = 6). Group 1 received standard rat feed, Group 2 was exposed to BLCO (0.1 mL/g of rat feed) for 3 weeks, and groups 3–5 were treated with zinc (50 mg/kg/day), vitamin E (400 IU/kg), or both [vitamin E (400 IU/kg) + zinc (50 mg/kg/day)], respectively for 1 week after BLCO exposure for 3 weeks. Locomotive, anxiolytic, depressive-like behaviours and spatial memory were assessed using the open-field test, elevated plus maze, forced swim test and Y-maze. Rats were sacrificed and the brain samples were collected for biochemical assays at the end of the behavioural tests.

**Results:** Zinc and vitamin E supplementation (individually or combined) significantly increased brain total antioxidant capacity and superoxide dismutase (SOD) activity, reduced inflammatory markers (TNF-alpha) and lipid peroxidation, normalized neurotransmitter levels in the brain and improved behavioural performance.

**Conclusion:** Treatment with Zn and/or vitamin E reverses BLCO-induced neurobehavioural alterations via modulation of oxidative stress, inflammation and neurotransmitters.

## 1. Introduction

Neuropsychological and neurodegenerative disorders, such as anxiety, depression, memory impairment and others, are on the rise and are influenced by multiple factors. Among these, exposure to neurotoxins is increasingly recognized as a significant contributor to the onset and progression of these disorders [1, 2]. The pervasive contamination of ecosystems by crude oil presents a significant environmental and public health challenge. This issue is particularly acute in regions with extensive oil exploration and production, where the deleterious effects of crude oil exposure on human and animal health are becoming increasingly evident. The Niger Delta region of Nigeria is primarily made up of agricultural communities where farming and fishing are the main sources of livelihood [3]. When oil and petroleum hydrocarbons degrade in aquatic habitats, they accumulate in fish and other seafood, as well as in farm produce from adjacent farmlands, eventually making their way to humans and animals [4]. This exposes the local population to high levels of toxic substances, which can have detrimental effects on their health.

While crude oil's combustible nature initially led to attempts at using it for illumination, its strong odour and fumes quickly rendered it unsuitable for lighting purposes [5]. Alarmingly, some communities in the Niger Delta continue traditional practices involving direct crude oil consumption. These practices, often based on misinformation, claim benefits like detoxification, anticonvulsant properties and treatment for skin inflammation [6]. Additionally, some communities use a mixture of crude oil and olive oil in traditional medicine [7]. Beyond direct consumption, coastal communities face significant indirect exposure to crude oil through their diet. Consuming marine life from polluted waters leads to the unintentional ingestion of oil contaminants [7]. Similarly, the use of crude oil for topical applications on burns, skin ulcers and wounds poses a health threat [6]. Perhaps most concerning is the situation in communities where polluted rivers are the only available source of drinking water [8]. These practices highlight a critical lack of awareness regarding the severe neurological damage that crude oil exposure can cause [6].

Crude oil's hydrophobicity, or its affinity for lipids, allows it to readily pass through cell membranes [9]. This disrupts cellular function and can lead to energy depletion and metabolic dysfunction. Exposure to its components has been linked to various health problems, including respiratory issues, headaches, dizziness, fatigue and reproductive disorders [10, 11]. Chronic exposure can lead to even more severe consequences, including abnormal heart rhythms, seizures and comas [12]. Studies have also linked crude oil to developmental problems in children, lower birth weight and damage to the liver and gastrointestinal system [13–15]. Of particular concern are the neurological effects of crude oil exposure.

Furthermore, the social and economic impacts of these disorders on affected individuals and their families cannot be ignored. Research suggests that crude oil components can disrupt brain development in infants [16] and alter neurotransmitter function, potentially leading to depression, anxiety and memory impairment [6, 9, 17, 18]. The prevailing theory behind these neurological effects centres on cellular damage and inflammation caused by crude oil's interaction with the body. Metabolism of crude oil components generates reactive oxygen species (ROS), which damage cells and trigger inflammation [19–21].

Vitamin E is a naturally occurring antioxidant found in plants [22], and its role in mitigating oxidative damage is well documented, and it has been implicated in the prevention of neurodegenerative diseases [23, 24]. Its extended lifespan in brain tissue compared to other vitamins' ability to scavenge free radicals and protect cell membranes positions it as a potential candidate for treatment [25–27].

Zinc has garnered attention for its neuroprotective properties. Zinc supplementation has also shown promise in reducing oxidative stress and enhancing cognitive function in older adults [28]. Zinc is a critical trace element involved in numerous biological processes, including neurotransmission and enzyme function [29, 30]. Beyond its antioxidant role, zinc is crucial for neurogenesis and immune function and modulating synaptic plasticity [31].

The connection between oxidative stress, neuroinflammation and neuropsychiatric disorders like anxiety, depression, and psychosis is well established [32]. Furthermore, imbalances in various neurotransmitters are known to contribute to these conditions [33]. Despite the known neurotoxic effects of crude oil and the potential benefits of readily available zinc and vitamin E, there is a paucity of research specifically addressing their efficacy in mitigating neurochemical changes and behavioural deficits induced by crude oil. Current studies have largely focussed on the effects of these supplements in different contexts, but their individual and synergistic potentials in a crude oil exposure model remain underexplored. The findings could have broader implications for regions affected by oil pollution, providing a scientific basis for public health interventions and policy decisions aimed at protecting vulnerable populations.

## 2. Materials and Methods

### 2.1. Chemicals

Bonny light crude oil (BLCO) was obtained from the Nigerian National Petroleum Corporation (NNPC) Portharcourt, Rivers State, Nigeria. ELISA kits for dopamine (Catalog No.: E-EL-0046), serotonin (5-HT) (Catalogue No.: E-EL-0033), glutamate (Catalog No.: E-EL-H6069), and TNF-alpha (Catalog No.: E-EL-R2856) were gotten from Elabscience, USA, while the acetylcholine kit (Catalog No.: STA-603) was purchased from Cell Biolab, Inc., San Diego, USA. Both zinc tablets and vitamin E capsules were purchased from Uche Care Pharmaceutical Store, an accredited and known dealer in pharmaceuticals in Ondo City, Ondo State, Nigeria.

### 2.2. Experimental Animal

Thirty (30) male albino rats weighing 160 ± 10 g and aged between 10 and 12 weeks were obtained from the animal house of the University of Medical Sciences, Ondo State. The animals were monitored under standard laboratory conditions: at 35.5°C–37.0°C and 12 h light: 12 h darkness cycle throughout the experiment, which allowed free access to standard rat pellets and clean water.

### 2.3. Experimental Design

Two weeks after acclimatization, thirty (30) male Wistar rats (160 ± 10 g) were assigned into five groups (*n* = 6). Group 1 received standard rat feed, Group 2 was exposed to BLCO (0.1 mL/g of rat feed) [34, 35] for 3 weeks, and groups 3–5 were treated with zinc (50 mg/kg/day) [36], vitamin E (400 IU/kg) [37], or both [vitamin E (400 IU/kg) + zinc (50 mg/kg/day)] respectively for 1 week after BLCO exposure for 3 weeks. The dosage of BLCO realistically mimics what might occur in environmental contamination scenarios. All drugs were administered daily via oral gavage between 7:30 a.m. and 9:30 a.m. Co-administration of Zn and vitamin E took place at a 60-min interval.

### 2.4. Sample Collection

After the experiment, the animals were euthanized by cervical dislocation. The brains were carefully dissected out, rinsed with ice-cold, freshly prepared phosphate-buffered saline (PBS, pH 7.4) to remove blood, and weighed before homogenization. Following this, brain tissues were homogenized in fresh PBS. The homogenates were then centrifuged at 4°C for 5 min at 10,000 rpm. Supernatants were collected immediately for subsequent biochemical assays and stored at −20°C or below.

### 2.5. Neurobehavioural Tests

Open field test (OFT), Y-maze test, elevated plus maze (EPM) test and forced swim test (FST) were the behavioural tests of interest in this study. All behavioural tests were recorded live using a camcorder and then scored manually by at least two independent trained observers who were unaware of the research design or the drug treatment. Anxiolytic, antidepressive-like, and cognitive-enhancing effects of zinc and vitamin E or their co-administration, on BCLO-induced neurobehavioural alterations were investigated using the Y-maze test FST on day 28, while neurobehavioural tests in the EPM and OFT were conducted earlier on day 27 at test intervals of approximately 3 hours to prevent stress on the animals. After each rat was assessed in OFT, EPM and Y-maze, the test arena was cleaned with 70% alcohol to eliminate olfactory bias, and the area was allowed to dry before introducing a fresh animal.

### 2.6. OFT

Before the OFT, the animals were acclimated to the test room for at least 40 min. Each rat was introduced singly into the open-field arena, and behavioural parameters, including total locomotion, rearing frequency, and grooming frequency, were recorded [38]. The test lasted for a total duration of 30 min, with observations recorded at 5-min intervals. The test was conducted in a single session without breaks, and all behaviours were monitored continuously during this time. The arena was cleaned between tests to prevent olfactory cues from affecting subsequent animals.

### 2.7. EPM

Rats' anxiety-like behaviour was evaluated using the EPM. Before the test, rats were kept in a silent room for at least 40 min. Each rat had 5 min to explore the apparatus freely after being placed in the centre of the EPM facing an open arm [38]. The time spent in the open and closed arms of the maze by each rat was recorded.

### 2.8. FST

The FST assessed depression or despair-like behaviour in rats. During this task, rats were positioned for 5 min inside an inescapable cylinder containing water at a temperature of 23°C, with a depth that prevented the rats from reaching the bottom and resting. The test focuses on observing how animals respond to the brief but unavoidable stress of swimming and adopt an immobile posture. In the present study, each rat was individually forced to swim after a pretest had been done 24 h before the main test [39]. The latency to immobility and the amount of time the rats spent being immobile were recorded.

### 2.9. Y-Maze Test

The percentage of alternation (%) and number of arm entries were the needed parameters used to assess short-term spatial memory [38]. The animals were maintained in the silent lab for at least 40 min before the Y-maze experiment. The rats were placed in the Y-maze for 5 min.

### 2.10. Biochemical Assays

Brain tissue homogenates were analyzed for various biochemical markers. Lipid peroxidation was assessed by measuring malondialdehyde (MDA) content using a colourimetric method. This method is based on the reaction of MDA with thiobarbituric acid (TBA) to form a pink-coloured MDA-TBA adduct, which is measured spectrophotometrically at 532 nm. The intensity of the colour produced is directly proportional to the MDA concentration in the sample, providing an index of lipid peroxidation [40]. Superoxide dismutase (SOD) was assayed using the Marklund and Marklund method [41], while total antioxidant capacity (TAC) was measured using a colourimetric assay that detects the reduction of copper ions [42]. Nitrite levels, a marker of nitric oxide production, were assessed using a modified Griess method [43]. Finally, neurotransmitter concentrations (dopamine, glutamate and serotonin) and tumour necrosis factor-alpha (TNF-*α*) were quantified using enzyme-linked immunosorbent assays (ELISA) according to the kit manufacturer's protocols.

### 2.11. Statistical Analysis

The study employed GraphPad Prism version 9.0.5 (GraphPad Software, San Diego, USA) for the analysis of the collected data. Data were shown as Mean ± Standard Error of Mean (SEM) for each group. One-way analysis of variance (ANOVA) was used to analyze the mean differences, and a Tukey post hoc test was used for multiple comparisons. A significance level of *p*  <  0.05 was used to determine statistical significance.

## 3. Results

### 3.1. Effect of Treatment With Zinc and Vitamin E on the Brain Weight of Rats Fed With BLCO-Contaminated Diet

**Figure 1:**
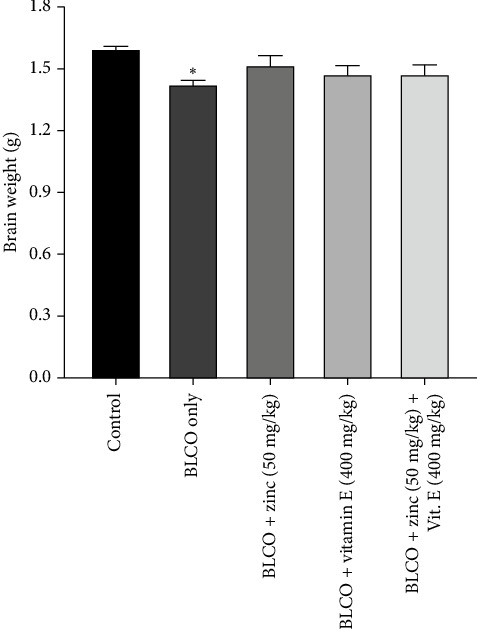
Effect of treatment with zinc and vitamin E on the brain weight of rats fed with a bonny light crude oil-contaminated diet. Bars represent Mean ± Standard Error of Mean (SEM) (*n* = 5) (one-way ANOVA followed by Tukey's post hoc test). ⁣^∗^means significantly different (*p* <  0.05) from the control.

The effect of the treatment on the brain weight of rats fed with a BLCO-contaminated diet is shown in Figure 1. Accordingly, there was a significant decrease (*p* < 0.05) in the brain weight of the BLCO-fed group relative to the control group. However, treatment with zinc only, vitamin E only, zinc and vitamin E produced no statistically significant difference (*p* > 0.05) in the brain weight when compared to BLCO-fed rats.

### 3.2. Effect of Treatment With Zinc and Vitamin E on the Neurobehavioural Patterns Observed in an OFT in Rats Fed With BLCO-Contaminated Diet

**Figure 2:**
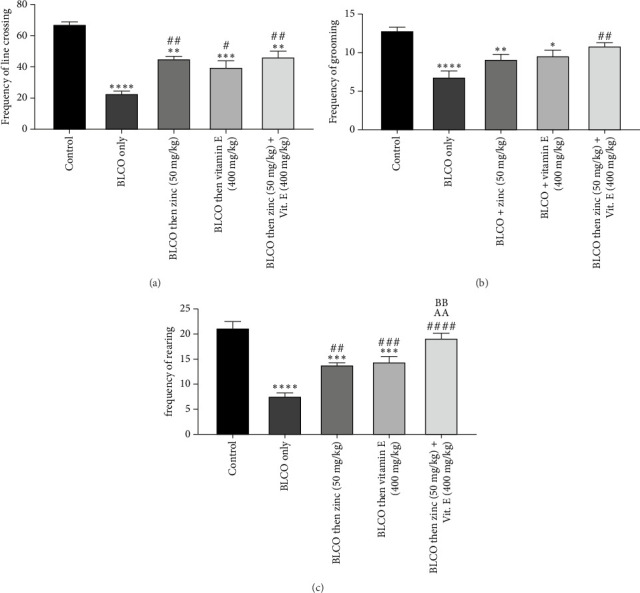
Effects of zinc or/and vitamin E on the (a) line crossing frequency, (b) grooming frequency and (c) rearing frequency of an open field test (OFT) in rats fed with bonny light crude oil-contaminated diet. Bars show the Mean ± Standard Error of Mean (SEM), with *n* = 6 (one-way ANOVA with Tukey post hoc analysis in between). ⁣^∗^p < 0.05, ⁣^∗∗^p < 0.01, ⁣^∗∗∗^p < 0.001 and ⁣^∗∗∗∗^p < 0.0001 significantly different relative to control; ^#^p < 0.05, ^##^p < 0.01 and ^###^p < 0.001, ^####^p < 0.0001 significantly different relative to BLCO only. ^AA^*p* < 0.01 and ^BB^*p* < 0.01 were significantly different compared to zinc only and vitamin E only, respectively.

As presented in Figures 2(a), 2(b) and 2(c), the effect of zinc or vitamin E on BLCO-induced locomotive decline was assessed based on the frequencies of line crossing, grooming, and rearing using an OFT. BLCO-fed rats (0.1 mL/g of feed) significantly (*p* < 0.05) reduced the locomotive parameters as indicated by reducing the total number of line crossings, rearings and groomings compared with the control group. However, treatment with zinc and/or vitamin E caused a significant (*p* < 0.05) increase in the locomotive parameters in the OFT when compared with the BLCO-fed rats. No significant difference (*p* >  0.05) was seen in the zinc vs. vitamin E treatment groups in all parameters. A significant increase (*p* < 0.05) in the rearing frequency was noticed in the zinc + vitamin E treatment group compared to zinc only and vitamin E only.

### 3.3. Effect of Treatment With Zinc and Vitamin E on EPM Test of Rats Fed With BLCO-Contaminated Diet

**Figure 3:**
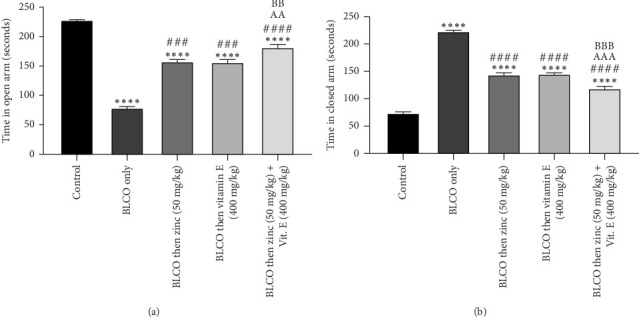
Effects of treatment with zinc or/and vitamin E on the duration spent in (a) open arms and (b) closed arms of the elevated plus maze in bonny light crude oil-fed rat. Bars show the Mean ± Standard Error of Mean (SEM), with *n* = 6 (one-way ANOVA with Tukey post hoc analysis in between) ⁣^∗∗∗∗^p < 0.0001 significantly different relative to control; ^###^p < 0.001 and ^####^*p* < 0.0001 significantly different relative to BLCO only. ^AAA^*p* < 0.001 and ^BBB^*p* < 0.001 were significantly different compared to zinc treatment only and vitamin E treatment only respectively.

As depicted in Figures 3(a) and 3(b), our findings reveal a notable (*p* < 0.05) reduction in the duration spent in the open arm and an elevation in the time spent in the closed arm during the EPM test, which assesses anxiety-related behaviour, among rats fed with BLCO (0.1 mL/g of feed) compared to the control group. Conversely, administration of zinc and/or vitamin E significantly (*p* < 0.05) extended the time spent in the open arm while reducing the time spent in the closed arm in comparison to the BLCO-treated group. While no significant difference (*p* >  0.05) was seen in the zinc versus vitamin E treatment groups in both time spent in the open arm and closed arm, there was a significant increase and decrease (*p* < 0.05) in the time spent in the open arm and closed arm respectively in zinc + vitamin E treatment.

### 3.4. Effect of Treatment With Zinc and Vitamin E on FST in Rats Fed With BLCO-Contaminated Diet

**Figure 4:**
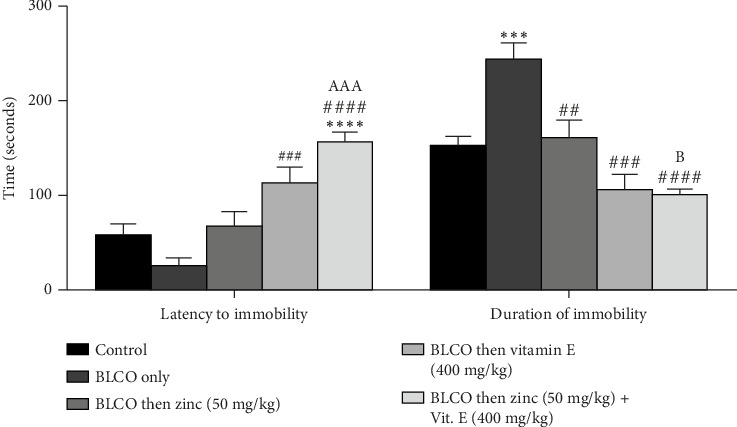
Effects of treatment with zinc and vitamin E on the latency to immobility and duration of immobility of forced swim tests of rats fed with bonny light crude oil-contaminated diet. Bars show the Mean ± Standard Error of Mean (SEM), with *n* = 6 (one-way ANOVA with Tukey post hoc analysis in between). ⁣^∗∗∗∗^p < 0.0001 indicates a significant difference from control; ^##^p < 0.01, ^###^p < 0.001 and ^####^p < 0.0001 indicates a significant difference from BLCO alone. ^AAA^*p* < 0.001, and ^B^*p* < 0.05 were significantly different compared to zinc treatment only and vitamin E treatment only, respectively.

As shown in Figure 4, the effect of zinc or vitamin E on depressive-like behaviour caused by BLCO exposure in rats was assessed and corroborated using the FST model. The duration of immobility significantly increased (*p* < 0.05), while there was no statistically significant difference (*p*  >  0.05) in the latency to immobility in BLCO-fed rats (0.1 mL/g of feed) when compared with the control group. Moreover, treatment with zinc and vitamin E elicited a significant (*p* < 0.05) decrease in immobility time and latency to immobility compared to BLCO-exposed rats. While no significant difference (*p* >  0.05) was seen in the zinc versus vitamin E treatment groups in both latency to immobility and duration to immobility, there was a significant increase (*p* < 0.05) in the latency to immobility of zinc + vitamin E treatment group compared to zinc only and decrease (*p* < 0.05) in duration of immobility in zinc + vitamin E treatment group compared to vitamin E only.

### 3.5. Effect of Treatment With Zinc and Vitamin E on Y-Maze Test in Rats Fed With BLCO-Contaminated Diet

**Figure 5:**
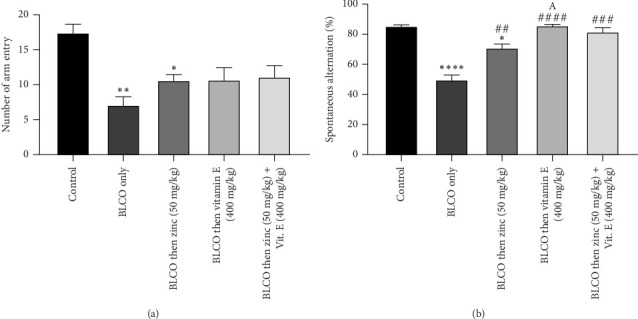
Effects of treatment with zinc and vitamin E on (a) the number of arm entries and (b) the percentage of spontaneous alternation in the Y-maze test in rats fed with a bonny light crude oil-contaminated diet. The bars show the Mean ± Standard Error of Mean (SEM) for *n* = 6 (one-way ANOVA with a Tukey post hoc test in between). Significant differences were observed between ⁣^∗^*p* < 0.05, ⁣^∗∗^*p* < 0.01 and ⁣^∗∗∗∗^*p* < 0.0001 in comparison to control; ^##^*p* < 0.01, ^###^*p* < 0.001 and ^####^*p* < 0.0001 in comparison to BLCO alone. ^A^*p* < 0.05 significantly different compared to zinc treated group.

As shown in Figures 5(a) and 5(b), the effect of zinc, vitamin E or a combination of both on the cognitive impairment caused by BLCO exposure in rats was assessed by Y-maze. The number of arm entries and percentage of spontaneous alternation significantly (*p* < 0.05) decreased in BLCO-fed rats (0.1 mL/g of feed) compared to the control group. However, zinc, vitamin E or a combination of both vitamin E treatments elicited a significant (*p* < 0.05) increase in spontaneous alternation compared to BLCO-exposed rats. A significant increase (*p* < 0.05) was noticed in the percentage alternation of vitamin E versus zinc treatment group.

### 3.6. Effect of Treatment With Zinc and Vitamin E on the Brain Oxidative Stress/Inflammatory Markers in Rats Fed With BLCO-Contaminated Diet

**Figure 6:**
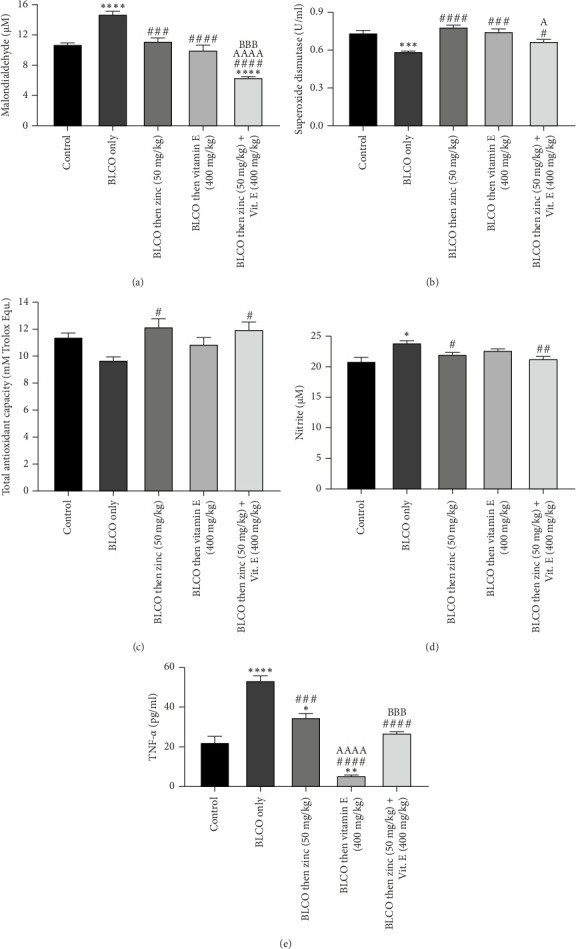
Effects of treatment with zinc and vitamin E on the brain (a) malondialdehyde level, (b) superoxide dismutase activity level, (c) total antioxidant capacity, (d) nitrite and (e) TNF-alpha in rats fed with bonny light crude oil-contaminated diet. Bars show the Mean ± Standard Error of Mean (SEM), with *n* = 5 (one-way ANOVA with Tukey post hoc analysis in between). Significant differences were observed between the groups: ⁣^∗^*p* < 0.05, ⁣^∗∗^*p* < 0.01, ⁣^∗∗∗^*p* < 0.001 and ⁣^∗∗∗∗^*p* < 0.0001 in comparison to control; ^#^*p* < 0.05, ^##^*p* < 0.01, ^###^*p* < 0.001 and ^####^*p* < 0.0001 in comparison to BLCO alone. ^A^*p* < 0.05, ^AAAA^*p* < 0.0001 significantly different compared to zinc treatment only and ^BBB^*p* < 0.001 significantly different compared to vitamin treatment E only respectively.

As presented in Figures 6(a), 6(b), 6(c), 6(d) and 6(e), the results showed that BLCO-exposed rats had a significant (*p* < 0.05) increase in the brain levels of MDA, nitrite, and TNF-alpha and a decrease in SOD with a relative to control. Conversely, when compared to rats fed BLCO, treatment with zinc or/and vitamin E resulted in a significant (*p* < 0.05) increase in TAC and SOD, as well as a significant drop in MDA, nitrite and TNF-alpha. A significant difference (*p* < 0.05) was observed in the TNF-alpha level of the vitamin E-only treatment group when compared to zinc only treatment. Similarly, there was a significant decrease and increase (*p* < 0.05) in the MDA and SOD respectively of the zinc + vitamin E treatment group compared to zinc only with a significant difference (*p* < 0.05) in the TNF-alpha zinc + vitamin E treatment group compared to vitamin E only as well.

### 3.7. Effect of Treatment With Zinc and Vitamin E on Neurotransmitter in Rats Fed With BLCO-Contaminated Diet

**Figure 7:**
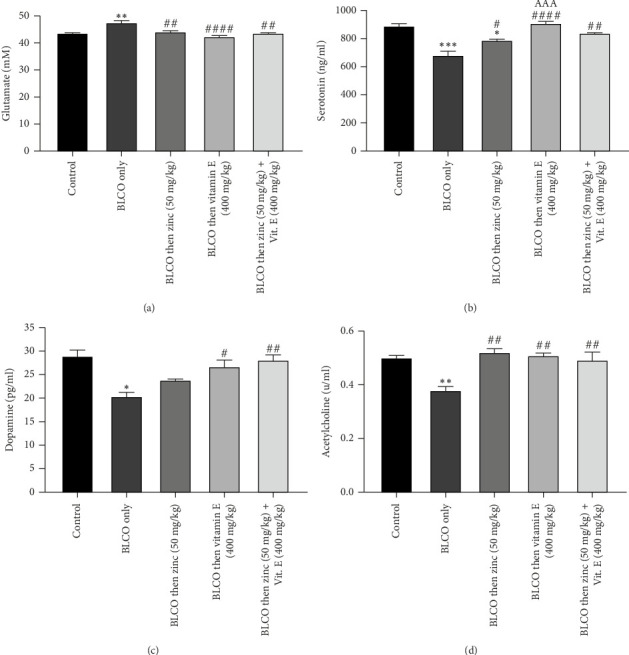
Effects of treatment with zinc and vitamin E on the brain (a) glutamate, (b) serotonin, (c) dopamine and (d) acetylcholine levels of rats fed with Bonny light crude oil-contaminated diet. The bars show the Mean ± Standard Error of Mean (SEM) for *n* = 5 (one-way ANOVA with a Tukey post hoc test in between). ⁣^∗^*p* < 0.05, ⁣^∗∗^*p* < 0.01 and ⁣^∗∗∗^*p* < 0.001 indicate significant differences from the control group; ^#^*p* < 0.05, ^##^*p* < 0.01 and ^####^*p* < 0.0001 indicate significant differences from the BLCO group alone. ^AAA^*p* < 0.001 significantly different compared to zinc treatment only.

As presented in Figures 7(a), 7(b), 7(c) and 7(d), the results showed that BLCO-exposed rats produced a significant (*p* < 0.05) decrease in the brain's levels of dopamine serotonin (5-HT), acetylcholine, and glutamate relative to the control. In contrast, compared to BLCO-fed rats, treatment with zinc or/and vitamin E resulted in a large (*p* < 0.05) rise in dopamine, serotonin and acetylcholine levels as well as a significant drop in glutamate. While no significant difference (*p* >  0.05) was seen in the zinc versus vitamin E treatment groups in the glutamate, dopamine and acetylcholine levels, a significant difference (*p* < 0.05) was observed in the serotonin level of vitamin E only treatment group when compared to Zinc only treatment. There was no significant difference (*p* > 0.05) in the levels of neurotransmitters in the zinc + vitamin E treatment group compared to zinc only and vitamin E only.

## 4. Discussion

Neurodegenerative diseases and psychiatric conditions are on the rise, with factors like genetic alterations, ageing, and environmental toxins playing a significant role [1, 44], and various environmental toxicants have been implicated in causing neuro-catastrophes on the peripheral nervous system (PNS) and the central nervous system (CNS), leading to neurotoxicity and behavioural deficits [1, 33]. A widely accepted theory suggests that most neurotoxins disrupt cellular function and trigger inflammation, leading to neurological damage. When metabolized by the body, crude oil components generate ROS. These ROS damage cells and initiate inflammatory responses [19, 20]. Consequently, oxidative stress and inflammation are well-established pathways to neurodegenerative diseases and mental health conditions [21].

Mitigating effects of zinc and vitamin E against neurotoxicity and behavioural deficits caused by crude oil exposure in rats was investigated in the present study. We employed a battery of behavioural tests (OFT, FST, EPM and Y-maze) to assess the effects of BLCO in rats. Our findings demonstrate that BLCO exposure significantly impaired behavioural performance in various tests.

The EPM test revealed anxiety-like behaviour in BLCO-treated rats, demonstrated by a significant decrease in time spent exploring the open arms. Anxiety disorders are associated with imbalances in neurotransmitters like serotonin, dopamine, and GABA [45]. The OFT assessed locomotor and exploratory behaviours. BLCO exposure significantly reduced grooming, rearing, and line-crossing frequencies, indicating decreased exploration and locomotion [38, 46].

Furthermore, the FST revealed and corroborated the depression-like behaviour in BLCO-treated rats as shown in the OFT. These rats displayed a significant decrease in latency to immobility (time spent actively trying to escape) and an increase in total immobility time. Finally, the Y-maze test revealed a significant decrease in spontaneous alternation in BLCO-fed rats. This suggests impaired short-term spatial working memory, possibly due to reduced cortical acetylcholine levels or disruption of acetylcholinesterase (ACh) activity by BLCO [47].

Chronic inflammation is associated with oxidative stress, which is defined as an imbalance between free radical formation (ROS) and antioxidant defences. It stimulates transcription factors, altering the expression of genes implicated in inflammatory pathways [48, 49]. Under normal conditions and the right concentration, the enzymatic antioxidant system, including SOD as well as antioxidant proteins, plays a critical role in neutralizing ROS [50, 51]. Normally, SOD disrupts superoxide radicals into hydrogen peroxide [52, 53]. In this present study, our results suggest not only a disruption in the antioxidant system but also a potential progression from oxidative stress to neuroinflammation in BLCO-fed rats, indicated by increased nitrite levels and TNF-alpha expression. Elevated nitrite levels in crude oil-exposed animals suggest blood-brain barrier dysfunction [54, 55]. Consequently, TNF-alpha gene expression, observed in our study, aligns with excitotoxicity and neuroinflammation reported by Olmos and Llado [56]. Notably, high TNF-alpha disrupts zinc homeostasis, leading to reduced synaptic activity and cognitive impairment [57], potentially explaining the behavioural alterations in BLCO-fed rats as shown in the results of this study. These findings suggest that BLCO disrupts the brain's antioxidant system.

Interestingly, treatment with vitamin E, zinc, or their combination attenuated lipid peroxidation and neuroinflammation. Vitamin E's mechanism likely involves its non-enzymatic antioxidant properties, scavenging free radicals [58]. This aligns with a similar study of [59], where vitamin E mitigated nicotine-induced behavioural and brain changes in rats. Zinc's influence is likely through stimulating the expression of enzymatic antioxidants and microglial cells, as evidenced by the significant increase in SOD activity [60, 61].

Neurotransmitters, released by neurons, are signalling molecules that facilitate communication between neurons and target cells. These molecules play a pivotal role in various neurobehaviours, such as cognition, emotional regulation, motivation and movement [62]. Exposure to toxins, such as BLCO, has been shown to alter these neurotransmitter levels, which can directly impact behavioural outcomes [63–65].

Our results demonstrate a significant increase in glutamate levels and a concomitant decrease in acetylcholine, dopamine and serotonin levels in the brain tissues following exposure to BLCO. This neurotransmitter imbalance is particularly concerning, as it suggests a disruption of normal brain function that could lead to behavioural changes. For example, elevated glutamate levels are known to be associated with excitotoxicity, potentially leading to neuronal damage and subsequent alterations in behaviour. Excessive accumulation of glutamate can induce oxidative stress, which leads to neuronal apoptosis and necrosis [52, 66, 67]. The observed increase in glutamate levels may contribute to the anxiety and depression-like behaviours noted in the behavioural tests, aligning with the glutamatergic theory of depression, which emphasizes the imbalance between excitatory glutamate and inhibitory GABA [66].

Moreover, the significant reduction in serotonin and dopamine levels corresponds with the monoaminergic theory of depression, which posits that deficiencies in these neurotransmitters contribute to depressive states [68]. The reduction of these key neurotransmitters following BLCO exposure may underpin the anxiety-like behaviours observed in the rats, reinforcing the notion that neurochemical disruptions lead to behavioural deficits.

In parallel, studies suggest that decreased levels of CNS serotonin, dopamine, and norepinephrine are associated with depression and anxiety-like behaviours following exposure to crude oil components [69]. The decrease in monoamines in our study aligns with findings from Kinawy [70], who reported similar reductions in various brain regions of rats exposed to gasoline, another crude oil component. This decrease corresponded with increased depressive, aggressive, anxiety and anger behaviours, thus strengthening the link between crude oil exposure and monoaminergic dysfunction.

BLCO is a complex mixture of hydrocarbons, including aliphatic and aromatic compounds, with polycyclic aromatic hydrocarbons (PAHs) being particularly concerning due to their neurotoxic potential. PAHs are lipophilic and can easily penetrate the blood-brain barrier, accumulating in brain regions such as the hippocampus, where they may disrupt normal neuronal function [9, 17]. In particular, PAH exposure has been linked to the inhibition of tyrosine hydroxylase (TH), the rate-limiting enzyme involved in the biosynthesis of catecholamines such as dopamine, norepinephrine, and epinephrine [33]. TH inhibition could lead to disruptions in neurotransmitter levels, contributing to the observed behavioural deficits, including locomotor impairments, as the hippocampus and basal ganglia, which are key regions involved in movement control, are highly sensitive to changes in amine biosynthesis [33]. In our study, we observed significant neurotransmitter alterations and behavioural deficits in BLCO-exposed rats, which align with the neurotoxic effects of PAH exposure described in the literature. The reduction in neurotransmitter levels and the associated behavioural impairments suggest that PAHs in BLCO may have contributed to the observed neurotoxic outcomes by disrupting dopaminergic and other catecholaminergic pathways in the brain [71–73]. These findings are consistent with studies showing that exposure to PAHs can lead to oxidative stress and inflammation, which further exacerbates neurodegenerative processes [17, 20].

Treatment with zinc and/or vitamin E significantly reversed these behavioural deficits, suggesting their potential anxiolytic and antidepressant properties. Although the exact underlying mechanisms remain unclear, these compounds may counteract the neurotransmitter disruptions induced by crude oil exposure. For instance, previous research by Szewczyk et al. [74] reported that zinc reduces depressive-like behaviour in rodents by modulating NMDA and AMPA receptors, which are critical for glutamatergic neurotransmission. Similarly, vitamin E's anti-inflammatory and antioxidant effects may help maintain neuronal integrity, potentially counteracting the stress mechanisms underlying anxiety and depression [53, 60].

Significantly, our research also demonstrated that zinc or vitamin E treatment, either individually or combined, effectively reversed the spatial memory impairment caused by BLCO exposure. This result implies a possible therapeutic function for zinc and vitamin E supplementation in counteracting BLCO-induced neurotoxicity could be in their modulatory roles on acetylcholine expression and metabolism. Previous studies reported a reversal of ACh activity in rats exposed to DEHP, another environmental toxin, after treatment with ZnSO_4_ [75] and humic acid treatment in ulcerative colitis-induced neurobehavioural alterations [38].

While the precise mechanisms through which these micronutrients provide their protective effects are yet to be determined, their capacity to combat oxidative stress and inflammation, both of which are associated with BLCO's neurotoxicity, offers a compelling explanation. In this study, both zinc and vitamin E demonstrated significant protective effects against oxidative stress, neuroinflammation, and behavioural deficits induced by BLCO exposure. However, when comparing their individual effects, vitamin E seemed to have a more pronounced impact on mitigating oxidative damage and neuroinflammation, likely due to its potent antioxidant properties and role in protecting cell membranes from lipid peroxidation [23–25]. Zinc, on the other hand, contributed to neuroprotection by enhancing antioxidant defences, reducing neuroinflammation, contributing to the expression of neurotransmitters, and supporting neurogenesis and synaptic plasticity [28–31].

When used together, zinc and vitamin E did not demonstrate a significant additive or synergistic effect. Nevertheless, their combination resulted in improved outcomes in reducing lipid peroxidation and enhancing SOD expression. These findings indicate that, although both compounds provide neuroprotective advantages, their effects might not be complementary in this situation. One reason for this lack of additive effect could be the similarity in their mechanisms, as both zinc and vitamin E are recognized for their ability to neutralize ROS and mitigate oxidative stress. Thus, it is conceivable that the protective properties of vitamin E alone were adequate to address the damage, rendering the supplementation of zinc unnecessary in certain aspects of neuroprotection.

Furthermore, the combined treatment did not exceed the individual effects of vitamin E in most areas of neuroprotection. This may suggest that vitamin E's function as a lipid-soluble antioxidant is primarily responsible for its protective effects against oxidative stress and neuroinflammation induced by BLCO. While zinc does provide additional benefits, its impact appears to be less pronounced than the significant membrane-stabilizing qualities of vitamin E.

It is also necessary to take into account that the lack of synergistic effect might stem from variations in their pharmacokinetics, bioavailability, or their specific roles within different cellular environments. Zinc plays an essential role in enzymatic functions and cellular defence systems, whereas vitamin E may more directly address oxidative stress in lipid-dense regions, such as cell membranes, where the components of BLCO are likely to cause the most harm.

The results of this study have significant implications for communities that traditionally use BLCO for medicinal purposes and are inevitably exposed to it through contaminated agricultural products and drinking water. In regions like the Niger Delta, where BLCO is consumed or used in folklore remedies for various health issues, the neurotoxicological findings of this research highlight the potential risks associated with BLCO exposure, including its ability to cause oxidative stress, neuroinflammation and behavioural impairments. Raising public awareness about the hazards of BLCO exposure and advocating for safer alternatives such as scientifically validated herbal remedies and easily accessible micronutrients like zinc and vitamin E could help reduce the long-term neurological effects associated with these traditional practices and exposures.

## 5. Conclusion

This research examined the possible neuroprotective properties of zinc and vitamin E in counteracting neurotoxic effects induced by BLCO in rats. Our results indicate that exposure to BLCO significantly impairs brain function, resulting in behavioural issues, oxidative stress, neuroinflammation, and changes in neurotransmitter levels. Notably, treatment with zinc or vitamin E, whether administered individually or together, successfully alleviated the negative effects caused by BLCO exposure. These results highlight the potential benefits of zinc and vitamin E supplementation in reducing BLCO-related neurotoxicity. Future research should investigate the specific mechanisms through which these micronutrients provide their protective effects.

## Data Availability

The data that support the findings of this study are available on request from the corresponding author. The data are not publicly available due to privacy or ethical restrictions.
